# Comment on: Indocyanine green fluorescence imaging to localize insulinoma and provide three-dimensional demarcation for laparoscopic enucleation: a retrospective single-arm cohort study

**DOI:** 10.1097/JS9.0000000000000983

**Published:** 2023-12-11

**Authors:** Youbao Huang, Dongyao Xu, Wei Wang

**Affiliations:** a Department of Hepatobiliary and Pancreatic Surgery, The Second Affiliated Hospital of Fujian Medical University, Quanzhou; b Fujian Medical University, Fuzhou, Fujian Province, People’s Republic of China

*Dear Editor,*

Fluorescence imaging with indocyanine green (ICG) has been used to assess the patency of reconstructed vessels, show lymphatic vessels requiring anastomosis, identify sentinel lymph nodes during breast and gastric tumor surgery, and visualize liver, pancreas, and gynecological tumors. Different from the obvious texture and temporal differentiation development caused by the hepatobiliary system involved in the full process of ICG uptake, metabolism, and excretion, pancreatic tissue and pipeline are only inertly involved in the in-vivo metabolic process of ICG, so the surgical fluorescence imaging of pancreatic tissue and pipeline is still in its infancy.

Recently, we read the article by Tao *et al*.^1^ in the *International Journal of Surgery* with great interest. The authors conducted a meaningful assessment of the feasibility and accuracy of Indocyanine green fluorescence imaging for intraoperative localization of insulinomas and margin assessment during laparoscopic insulinoma enucleation. For this article, we have the following comments:

First, according to the enrollment criteria, 11 patients were enrolled, and two patients were intraoperative pancreaticoduodenectomy and distal pancreatectomy. What was the tumor condition of these two patients, and why the operation mode was changed intraoperatively.

Second, in the paper, there is no distance from the tumor to the surface of the pancreas. In the study, two out of eight patients could not show the dynamic perfusion of ICG because the tumor was too deep, and the depth of the two tumors from the surface of the pancreas. Research^2^ shows that in non-invasive tumors, 100% of pancreatic neuroendocrine tumors were visualized by the first window (intraoperative 25 mg of ICG injection). After 1 mg ICG, whether the two patients failed to capture ICG dynamic perfusion, we have added more doses to see if the fluorescence developed and whether the author can supplement this information for each patient for our subsequent reference.

Third, the operation was done smoothly and brilliantly for the representative cases. A drainage tube was placed during the operation, whether the postoperative drainage fluid detected amylase and bilirubin index, and when the drainage tube was removed.

Despite these aforementioned points that need to be discussed, we are genuinely grateful for the authors’ work in writing this article. The results show that ICG fluorescence imaging during laparoscopic insulinoma enucleation adds a sharp tool for the surgeon and reduces postoperative pancreatic fistula or biliary leakage. However, in the future, we still need to explore the dosage, timing, staining method, and observation method of pancreatic fluorescent staining and further prove that these methods can benefit patients.

## Ethical approval

Not applicable.

## Consent

Not applicable.

## Sources of funding

This study did not receive any funding.

## Author contribution

Y.H., D.X., and W.W.: designed and wrote this letter.

## Conflicts of interest disclosure

There are no conflicts of interest.

## Research registration unique identifying number (UIN)

Not applicable.

## Guarantor

Wei Wang.

## Data availability statement

Not applicable.

## Provenance and peer review

Not invited.
